# Depression and decision-making capacity for treatment or research: a systematic review

**DOI:** 10.1186/1472-6939-14-54

**Published:** 2013-12-13

**Authors:** Thomas Hindmarch, Matthew Hotopf, Gareth S Owen

**Affiliations:** 1 King’s College London, London, UK; 2 Department of Psychological Medicine, Institute of Psychiatry, King’s College London, Weston Education Centre, London SE5 9RJ, UK

**Keywords:** Depression, Depressive disorder, Depressed, Decision-making, Informed consent, Competence, Mental competency, Mental capacity

## Abstract

**Background:**

Psychiatric disorders can pose problems in the assessment of decision-making capacity (DMC). This is so particularly where psychopathology is seen as the extreme end of a dimension that includes normality. Depression is an example of such a psychiatric disorder. Four abilities (*understanding*, *appreciating*, *reasoning* and ability to *express a choice*) are commonly assessed when determining DMC in psychiatry and uncertainty exists about the extent to which depression impacts capacity to make treatment or research participation decisions.

**Methods:**

A systematic review of the medical ethical and empirical literature concerning depression and DMC was conducted. Medline, EMBASE and PsycInfo databases were searched for studies of depression and consent and DMC. Empirical studies and papers containing ethical analysis were extracted and analysed.

**Results:**

17 publications were identified. The clinical ethics studies highlighted *appreciation* of information as the ability that can be impaired in depression, indicating that emotional factors can impact on DMC. The empirical studies reporting decision-making ability scores also highlighted impairment of *appreciation* but without evidence of strong impact. Measurement problems, however, looked likely. The frequency of clinical judgements of lack of DMC in people with depression varied greatly according to acuity of illness and whether judgements are structured or unstructured.

**Conclusions:**

Depression can impair DMC especially if severe. Most evidence indicates *appreciation* as the ability primarily impaired by depressive illness. Understanding and measuring the *appreciation* ability in depression remains a problem in need of further research.

## Background

Although jurisdictions vary in the wording of the abilities adult decision-makers are deemed to need in order to decide for themselves about treatment, an influential model has been outlined by Grisso & Appelbaum [1]. The model consists of four abilities.

1. *The ability to****express a choice***

2. *The ability to****understand****information relevant to treatment decision making*

3. *The ability to****appreciate****the significance of that information for one’s own situation, especially concerning one’s illness and the probable consequences of one’s treatment options; and*

4. *The ability to****reason****with the relevant information so as to engage in a logical process of weighing treatment options*

The four abilities model can be used as a guide in both clinical and legal capacity judgments. Without the sufficient possession of any one of these four abilities, a patient may be considered unable to exercise their autonomy in relation to treatment decisions.^a^ Suitably modified, this applies to other decisions such as research participation. There is a distinction between *impairment* of these decision-making abilities, which lie on continua and the *judgment* of DMC, which is binary. The DMC decision is ultimately normative and justifies allowing other stakeholders to make decisions on behalf of the patient.

Psychiatric disorders are risk factors for loss of DMC [2] but there is some debate about whether current models of decision-making capacity are able to accommodate the changes of emotion and value that can be seen in psychiatric disorders where understanding ability is often not impaired [3-6]. There is also the question of how much ability a patient requires to yield a categorical judgment of DMC [7] and the reliability of physician judgments [8]. Depression is significant in relation to these debates because of its high prevalence in health settings; its emotional nature; its dimensionality; and the often “understandable” quality of patient decision-making, which is perhaps best characterized by Appelbaum & Roth [9]:

“Of all the psychopathological processes associated with refusal [of treatment], depression is the most difficult to recognize, because it masquerades as, ‘Just the way I would think if it happened to me’ … The depressed patient is frequently able to offer ‘rational’ explanations for the choices that are made.”

If depression can impair DMC, then depressed individuals may be at risk of making treatment decisions without the abilities needed whilst being considered autonomous. The consequences of this are potentially disastrous; consider the case of John Smith, a man with progressive but nonetheless treatable respiratory failure and recognized severe depression. He refused medical treatment, dying without any attempt to cure his depression, despite the fact this known mental illness may well have altered his judgment and consequent DMC [10]. In such cases, ethical concerns arise about the protection of patient welfare.

We aimed to conduct a systematic review of medical and medical ethical research on depression and DMC. Our primary aims were (1) to review clinical ethical accounts of how DMC may be affected by depression and (2) to review the empirical literature and their measures of the impact of depression on DMC.

## Methods

A search was carried out attempting to locate all the literature relevant to the aims of the review. Inclusion criteria required that selected papers were (1) in the English language; (2) included significant clinical ethical analysis or contained relevant observational data using DMC assessment tools in depressed individuals. Papers were excluded if they: (1) were in a foreign language; (2) did not contain empirical data relating to the legal/ethical standards of DMC; or (3) lacked substantial ethical analysis.

Relevant articles were selected by TH and GO through a systematic search of electronic databases. These comprised MEDLINE (1946 to February 2012), PsycINFO (1946 to February 2012) and EMBASE (1974 to February 2012).

Search terms included: 1) Depression, Depressive disorder, Depressed and 2) Decision-making, Informed consent, Competence, Mental competency, Mental capacity.

These search terms were combined (1. Ω 2.) in keyword searches of all three databases. Titles and abstracts were screened for eligibility and where relevant, studies were read in through to determine eligibility. Citations of eligible articles were also screened.

All studies with empirical data were included that used measures of DMC related to legal/ethical standards and involved treatment or research decisions.

Making a distinction within the non-empirical literature between studies with and without “substantive” ethical analysis may seem subjective, but was readily achievable in practice. Non-data papers were only included if they addressed the *relation* between depression and DMC using ethical constructs and reasoning. Many utilised case studies in illustrating this. Papers that were not included typically involved rehearsals of extant law or pragmatic considerations in specific clinical scenarios such as constraints of time or risk assessment. Papers excluded by TH were reviewed by GO and no disagreements were found.

## Results

**Figure 1:**
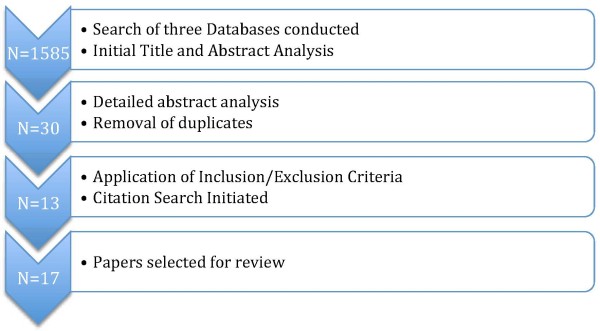
Summary of review methods.

The search produced a total of 1585 papers (PsycINFO = 121, MEDLINE = 1299, EMBASE = 165). The titles of all articles generated were read and examined on the above inclusion and exclusion criteria, with abstracts of possible importance considered for inclusion. After removal of duplicates and further analysis of abstracts, an initial total of 30 articles were selected and read in full. Application of inclusion/exclusion criteria narrowed this to 13 in the final review. The search was augmented through examination of cited material within the selected papers for further relevant material suitable for inclusion. The citation search produced a further 2 articles suitable for inclusion, bringing the final total of articles to 15. Additionally, contact with fellow researchers in the field highlighted 2 further papers by Bean et al. which were also included (Figure 1).

The heterogeneous methods used within the 17 included papers made systematic quality assessment of the papers unsuitable for the review, but all studies were found to relate to legal standards pertinent to the review. Two studies were finally excluded because they only related to supported decision making rather than to DMC (See Tables 1 and 2).

**Table 1:** Results from clinical ethical analyses

**Author & year**	**Source material used (e.g. *Clinical experience *** **or *case study)***	**Significant findings**
**Elliot (1997)**[11]	*Clinical experience.* Elliot integrates his personal experience and reasoning with that of other published literature.	When depressed research subjects cannot be held accountable for their treatment decisions, they lack capacity. Competency requires decisional authenticity and a minimal concern for one’s own welfare.
**Halpern (2010)**[12]	*Case study.* An example case is used as the foundation in explaining ‘Concretized Emotion-Belief Complexes’.	Depression may lead to ‘Concretized Emotion-Belief Complexes’ where patients hold rigid beliefs such that they are unable to engage in *appreciation.* Emotional and cognitive factors influence capacity.
**Leeman (1999)**[10]	*Case study.* Two case vignettes, those of John Smith an Elizabeth Bouvia are considered in relation to capacity.	Depression may cause a breakdown in the *appreciation* component of mental capacity, but assessing this clinically poses a difficult task.
**Meynen (2010)**[13]	A *phenomenological* conceptual argument founded on other academic literature.	Depression renders an individual unable to *appreciate* future possibilities.
**Rudnick (2002)**[14]	*Case study.* An anonymised case vignette serves to illustrate and articulate the way depression can impair capacity.	Depression can render an individual lacking capacity, despite leaving cognitive components of capacity in tact. A consideration of emotion should be integrated into capacity assessment.
**Sullivan & Youngner (1994)**[15]	*Review article.* Academic literature both medical and legal is amalgamated with a number of *case studies* featured.	Depression produces subtle distortions of decision making that are difficult to detect. Decisions made by depressed individuals may seem reasonable. Depression and its severity does not necessarily impede decision-making. The *appreciation* component of depression must be assessed carefully, as depression may impair appreciative ability. Symptoms of depression can readily interfere with *understanding, appreciation* and *reasoning* ability.
**Young E.W. (1993)**[16]	Four *case studies* demonstrate the deficits in capacity that may manifest in depression, with comments from the perspective of the psychiatrist, the ethicist, and the legal counsel.	Depression can impair *appreciation.* Determination of competence requires that multiple factors are considered including a consideration of patient circumstances, their familial perception of the situation, reversibility of the patient’s emotional state and cognitive functioning.

**Table 2:** Results from Empirical Studies

**Author & year**	**Participants**	**Depression type/symptom severity**	**Decision**	**Measures used**	**Study objectives**	**Significant findings**
**Appelbaum et al. (1999)**[17]	26 female outpatients pre-selected on basis that they were able to communicate.	DSM–IV major depression.	Research participation	MacCAT-CR scores on understanding, reasoning and appreciating abilities.	Assessment of decision-making abilities to consent to research.	Most subjects performed well on ability measures, maintaining this over the course of their admission. Low scores in appreciation and reasoning measures were recorded in a small subgroup.
		HDRS				Mean scores for: understanding 23.33 (out of max 26); appreciation 4.89 (out of max 6); reasoning 6.5 (out of max 8).
		Mean Score = 18				
		Range = 15-25				
**Cohen et al. (2004)**[18]	20 psychiatric inpatients	DSM-IV major depression.	Research participation	MacCAT-CR scores on understanding, reasoning and appreciating abilities.	Assessment of decision-making abilities to consent to 2 research protocols with different risks.	Subjectstended to score in the highest range of all three abilities for both research protocols. The poorest scores were in reasoning but with 90% scoring above the mid point of the reasoning scale in the high-risk study.
		BDI				
		Mean score = 41				
		SD = 9.5				
**Bean et al. (1994/1996)**[19,20]	96 psychiatric inpatients referred for ECT	‘Major depression unresponsive to medication’ (Personal correspondence from author). Depression type and symptom severity not given in paper.	Treatment participation	Competency Schedule (CIS) scores (a 15 item questionnaire which the authors try to map to 4 standards: evidencing a choice, understanding the issues related to treatment, evidence for a rational reason for the choice, appreciation of the nature of the situation).	To compare physicians’ judgements of competency with scores on the CIS.	Complex presentation of findings. Physician judgments of competency in depressed patients awaiting potential ECT matched well with CIS scores. The item on the CIS that assesses a patients’ ability to specify (or know) the potential benefits of treatment is the best single discriminator of physician judgement (Wilk’s Lambda = 0.49). The authors map this ability to “understanding the issues related to treatment” though, in MacCAT terms, it would map to appreciation: acknowledgement of potential benefit of treatment.
				Unstructured physician judgments of competency.		21 patients (21.9%) were categorized by the physician as unable to give consent for ECT.
**Grisso & Appelbaum (1995)**[21]	92 psychiatric inpatients	DSM-IV Major depression. (Severity not reported)	Treatment participation	Pre-cursor instruments to MacCAT-T. Scores on understanding, appreciation and reasoning abilities.	Assessment of decision-making abilities to consent to treatment.	Most subjects scored well on all abilities. Appreciation was most impaired ability. Subjects with scores indicating impairment in: understanding n = 5, (5.4%); appreciation n = 11 (12.0%); reasoning n = 7 (7.6%).
						Compound measures: understanding and/or appreciation n = 17 (18.5%); understanding and/or reasoning n = 11 (12.0%); appreciation and/or reasoning n = 17 (18.5%); understanding, appreciation and/or reasoning n = 22 (23.9%).
**Lapid et al. (2003)**[22]	40 psychiatric inpatients referred for ECT	DSM-IV major depression including unipolar, bipolar and schizoaffective depression.	Treatment participation	MacCAT-T scores on understanding, appreciation, reasoning and expressing a choice.	Assessment of decision-making abilities to consent to treatment before and after standard and experimental educational intervention.	Subjects scored well both before and after both standard and experimental interventions and both educational interventions increased scores somewhat.
		HDRS				A subgroup of patients with psychotic symptoms (n = 11) scored lower on the appreciation subscale compared with the nonpsychotic group (p < 0.001). The lowest appreciation score was 2 (scale range 0-4) indicating that no subject scored less than the mid-point of the scale.
		Mean =30.35 and 31.30. Range 21.0- 47.0 and 14.0 -42.0. (These apply to the standard and experimental intervention groups respectively).				
**Owen et al. (2008)**[2]	67 psychiatric inpatients	ICD-10 depression (Severity not reported)	Treatment participation	Structured clinical judgment using the MacCAT-T.	Determine prevalence of DMC for treatment in psychiatric inpatients with depression.	31% lacked DMC for treatment (medication or hospital care) - 95% CI 20-44.
**Owen et al. (2009)**[23]	64 psychiatric inpatients	ICD-10 non-psychotic disorders Depression =46 Post Traumatic Stress Disorder = 3 Personality disorder = 15 (Severity not reported).	Treatment participation	Structured clinical judgment using the MacCAT-T.	To investigate clinical associations with DMC in depressed patients.	Insight in non-psychotic disorders like depression (as opposed to psychotic disorders like schizophrenia, mania) was a poor “test” of DMC.
				Insight measured using the SAI-E		ROC analysis gave an AUC of 0.86. Sensitivity 1.00, specificity 0.44.
				Depressed mood using the BPRS		Severity of depressed mood associated with DMC with large effect size (Hedges’ g 1.25; 95% CI 0.64--‒1.85).
**Owen et al. (2011)**[24]	Mixed group of psychiatric inpatients	ICD-10 Schizophrenia and related disorders = 40 Depression = 16 (Severity not reported).	Treatment participation	Structured clinical judgment using the MacCAT-T.	To investigate the association between depression and regaining DMC following 1 month of inpatient psychiatric treatment.	Compared with schizophrenia and related disorders depression was associated with a higher chance of regaining DMC for treatment (OR 5.35, 95% CI 1.47–9.55).
**Vollman et al. (2003)**[25]	35 psychiatric inpatients	ICD-10 Moderate/Severe Depression HDRS: Mean = 21.8.	Treatment participation	MacCAT-T scores on understanding, appreciation and reasoning.	To investigate the competence of patients with depression to make treatment decisions.	Most subjects scored well on all abilities. Appreciation was most impaired ability. Subjects with scores indicating impairment in: understanding n = 5, (5.4%); appreciation n = 11 (12.0%); reasoning n = 7 (7.6%).
				Unstructured physician Judgment		Compound measures: understanding and/or appreciation n = 17 (18.5%); understanding and/or reasoning n = 11 (12.0%); appreciation and/or reasoning n = 17 (18.5%); understanding, appreciation and/or reasoning n = 22 (23.9%).
						One patient with depression (2.9%) was categorized by the physician as unable to give consent for drug therapy.

MacCAT = Macarthur Competency Assessment Tool, DMC = Decision-making Capacity, SAI-E = Expanded Schedule for the Assessment of Insight, BPRS = Brief Psychiatric Rating Scale, HDRS = Hamilton Depression Rating Scale, BDI = Beck Depression scale, ROC = Receiver Operating Characteristics, AUC = area under the curve, ICD-10 = International Classification of Disease 10, DSM = Diagnostic and Statistical Manual of Mental Disorders IV.

Analysis of the material revealed a number of themes, which are presented in a narrative manner below.

We found 7 papers using ethical analysis of clinical case histories &/or acquired clinical experience.

### Depressed patients lack appreciation

Several of the articles highlighted *appreciation* as the component of capacity lacking in depressed individuals. Grisso and Appelbaum in their four ability model give the following characterisation of appreciation; *‘[We] use the term in reference to people who, because of cognitive deficits or emotional states, fail to accept the relevance of their disorders or potential treatment consequences for their own circumstances.*’[1]. In simple terms, the transition from simply understanding the medical facts, to actual application of those facts by an individual to their own situation, can be impaired in the depressed individual.

Leeman (1999) used two case studies involving end of life decisions in highlighting where lack of *appreciation* rendered an individual incompetent. He used two examples (including that of John Smith cited above) in demonstrating the importance of distinguishing *understanding* and *appreciation*. He argued for a distinction between factually restating the consequences of a treatment decision and consideration of the situation-dependent, personal implications of that decision. John Smith, he contended, required an assessment of these latter abilities.

Young et al. (1993) also explored mental capacity in depressed patients via means of case studies in which a request to die is made. These cases again illustrate scenarios when depression was seen to impair a patient’s ‘*ability to comprehend the consequences of a choice*’ , again indicating that appreciative ability is at stake in depressive illness.

Meynen (2011) approached the topic from a phenomenological perspective to highlight the insufficiencies depressed patients have in their appreciative abilities. According to Meynen, mentally competent individuals possess an online perception of the world as a network of possibilities, from which further possibilities extend exponentially. The perception of these possibilities is intimately related to emotion and mood, and affective disorders may give rise to distortions. In other words, judgments concerning treatment benefits and risks, and their personal consequences, may, in depression, become founded on a skewed and altered perception of future possibility and compromise appreciative ability.

Halpern (2012), like Leeman and Young et al., utilized a case study in illustrating the deficits in appreciative capacity some depressed individuals have. Through the case of Ms. G, she introduced the concept of ‘concretized emotion-belief complexes’ , whereby a person is so certain that their future contains a certain set of events (as a result of an emotional extreme) that they cannot truly appreciate the benefits or risks involved in their treatment decision. Under her model, emotions and cognitive factors are implicitly linked and so are able to interfere with each other. She goes on to explain that although a person in this state can engage in apparent deliberation, these thought processes are overwhelmingly characterized by the rigid conviction of their current belief as perceived from their fixed emotional view. True deliberation and *appreciation* requires that an individual ‘*think[s] through alternatives*, and this thinking through alternatives needs to be *responsive to evidence’ .* (Authors’ italics).

#### Accountability

Elliot (1997) addressed the capacity of depressed individuals from a different perspective, drawing moral responsibility into the picture. He set the threshold of capacity at the point where a patient can be considered *accountable* for their choice. Whilst accepting DMC could be influenced by both cognitive and emotional factors, he pinpointed two criteria required for accountability that are potentially compromised in depressed patients; namely, decisional authenticity and a minimal level of concern regarding one’s own welfare. Lack of ‘decisional authenticity’ refers to the fact that depressed individuals may not make decisions that reflect their ‘true’ autonomous self. Equally, they may not possess a minimal level of concern for their own welfare, without which any claim of DMC is jeopardized. Even when *understanding* and *appreciative* ability remain intact, Elliot argued, depressive illness may leave an individual with so little self concern regarding the potential negative outcomes of a research decision, that they cannot be considered accountable for their decision to participate. This, Elliot said, amounts to not caring about risks rather than not appreciating risks. Without decisional authenticity or minimal self-concern, patients cannot be considered *accountable* for their actions, as they are a manifestation of the affective disorder rather than the truly autonomous individual that exists behind the façade of illness.

#### Emotion, cognition and capacity

All the clinical ethical literature recognizes interplay between cognition, emotion and DMC. The widespread view is that capacity assessment must take into account both cognitive and emotional factors. An extract from Sullivan & Youngner’s (1994) review demonstrates how the (emotional) symptoms of depression (**in bold**) might correlate with deficits in the cognitive (*understanding*, *appreciation* and *reasoning)* components of mental capacity:

*‘Depressive* ***helplessness*** *produces an underestimation of one’s possible effectiveness in the face of serious illness.* ***Guilt*** *and* ***worthlessness*** *may make one believe that suffering and death are deserved…* ***Anhedonia*** *may make it impossible to imagine that life will offer any pleasures for which it is worth enduring … illness. Depressive* ***hopelessness*** *can make it impossible to imagine that life will ever offer a better balance of pleasure and pain that it does at present.’*

Rudnick (2002), however, argued that a degree of separation between emotion and cognition exists; suggesting that coherence of personal preference may be disrupted even when the four abilities that make up standard assessment of DMC remain unscathed by depression. He, like others, proposed a strategy by which depressed patients can be protected against their own treatment decision. By asking previously depressed individuals their preferences regarding treatment of any future episodes of depression, and matching these to treatment preferences expressed during the depressive period, DMC may be better assessed and autonomy better respected. However, he did concede that in the initial episode, depressed individuals should be treated in their ‘best interests’ until restored to health. This matter is discussed later.

### MacCAT and other measures of DMC

Does depression impair performance on tests of decision-making capacity?

One of the earliest studies (Grisso & Appelbaum 1995) investigated hospitalised patients with major depression. A semi-structured interview was used to measure three of the four components of capacity to decide treatment (This was a precursor to the MacArthur Competence Assessment Tool – Treatment “MacCAT-T”), namely understanding, appreciation and reasoning ability. Of the ninety-two depressed participants, 23.9% (22/92) displayed an impairment in one or more of the assessed components, with half of these displaying an impairment in appreciation.

All but one of the included empirical data studies conducted after this date used the MacCAT and the four-ability model in measuring mental capacity. (Table 3).

**Table 3:** **The MacArthur competency assessment tool**[1]

	
1	A tool designed to help clinicians ‘obtain and organize information about patients’ decision-making abilities.’
2	Structured interview following fixed topics.
3	Flexible for use in ‘assessing patients with a wide range of illness, including psychiatric disorders.’
4	‘Used to assess the degree to which patients are Understanding the information and recognizing (Appreciating) the relevance of the information for their own situation. MacCAT-T then guides clinicians to explore how patients are thinking … so as to arrive at a picture of their reasoning abilities.’ Additionally, it assesses the ability to express a choice.
5	The assessment maps onto a quantitative rating system that allows objective scoring of a patients abilities.

The MacCAT only serves to identify deficits in decision-making capacity rather than to determine competence, although the former may influence the latter.

Three studies included MacCAT data. In their 1999 study, Appelbaum et al. (1999) measured the MacCAT abilities of moderately depressed inpatients to consent to clinical research, modifying the MacCAT for this purpose (MacCAT-CR). Capacity measurements were made within one week of admission with a second assessment made 8–10 weeks later. Almost all subjects performed well on initial capacity measures, maintaining this over the course of their admission. Similar findings are reported by Cohen et al. (2004).

In Lapid et al. (2003) forty severely depressed patients (DSM-IV criteria) were measured on MacCAT-T abilities to consent to electro-convulsive therapy (ECT). Judgements of DMC were not made. Overall, participants displayed good baseline scores on the MacCAT abilities at initial measurement including appreciation. 11 patients (27.5%) had active psychotic symptoms at the time of interview. This group had lower scores on the appreciation subscale compared with the non-psychotic population but no statistically significant differences in understanding, reasoning, and choice. As part of the study, Lapid et al. trialed a standard and an experimental educational intervention, finding that both improved MacCAT ability scores, though ceiling effects made interpretation difficult.

Vollmann et al. (2003) looked at MacCAT-T scores in depressed inpatients and the separate judgment of a physician, who was instructed to judge capacity ‘*freely on the basis of his or her own clinical experience*.’ Using various combinations of cut offs scores for understanding, appreciation and reasoning to define “impairment”, a range of impairment from 2.9% to 20% was found. Within the same sample, the attending physician judged only one patient as lacking in DMC.

Owen et al. (2008) conducted a study involving 350 consecutive admissions to a psychiatric hospital 67 of which made up a depressive subset. The study aimed to assess the prevalence of impaired DMC using the MacCAT-T to structure a clinical judgment. The prevalence of incapacity was estimated to be 31% in the depressed subset. Severity of depressed mood associated with lack of DMC.

Bean et al. (1994/6) developed a 15-item semi-structured Competency Interview Schedule (CIS) to measure DMC. It is discussed in two papers where the DMC of 96 inpatients to consent to ECT was assessed. The majority of patients had major depressive illness. All participating subjects had their decisional capacity judged by their attending physician before undertaking the CIS and 21.9% were judged lacking capacity to decide whether to opt for/against ECT. The closest correlation between item scores and physician judgment of DMC was that requiring the patient to specify the potential benefits of the treatment.

### Insight and DMC

Owen et al. (2009) further analysed the sample described in Owen et al. (2008). Two hundred participants in the study were subject to several assessments before judging DMC, including the MacCAT-T, the Expanded Schedule for the Assessment of Insight (SAI-E) and the Brief Psychiatric Rating Scale (BPRS). The relationship between insight (awareness of illness) and DMC was evaluated and found to be dependent on the disorder encountered. Whilst insight mapped to DMC well in illnesses like schizophrenia and mania, it mapped poorly in depressive illness (poor sensitivity) suggesting insight is not a good clinical indicator of DMC in depression. Severity of depressed mood associated strongly with DMC.

### Regaining DMC

Owen et al. (2011) using the above cohort also looked at which broad diagnostic groups associate with regaining DMC. One month following admission, patients with depression were found to be more likely than patients with schizophrenia and related disorders to regain DMC indicating that change, or *fluctuation,* of DMC is more a feature of depression.

## Discussion

This systematic review of the literature suggests that depression can impair DMC by impacting on *appreciation* and to a lesser degree, understanding and reasoning ability. Much depends upon illness severity and population studied. The literature is small and heterogeneous and so conclusions can only be tentative.

Clinical ethical analysis stresses appreciative ability as site of impairment. Different formulations of this inability exist in the literature and each is relatively unspecified. Two concepts seem to offer useful leads: Meynen (2010), Halpern (2010), and Sullivan & Youngner (1994) highlight an *inability to appreciate future possibilities* and Elliott (1997) highlights an *inability to maintain a minimal concern for self*. Both are viewed as normative inabilities that may threaten DMC. The affective symptoms of depression can theoretically distort [15] or blind [12] an individuals’ perception of the future, but as Meynen remarks, the *ability to appreciate future possibilities* needs, further specification and empirical testing [13]. Elliot presents the *minimal concern for self* concept as distinct from appreciation but this distinction depends upon the boundaries one places around the appreciation ability which seems sufficiently broadly defined to encompass minimal concern for self. It is also unclear how minimal concern for self differs from the trait of low self-esteem, which is common and is not diagnostic [26], and does not automatically confer lack of DMC.

The emphasis being put on appreciation in the clinical ethical studies is partly reflected in the empirical studies involving the MacCAT but it is weaker than the clinical ethical analysis would lead one to expect. Part of this may be explained by selection of cases with the clinical ethical analysis focusing on exceptional cases whilst the empirical studies take mixed populations of patients with varying levels of depression and different decisions (research and treatment). However, in the Lapid et al. (2003) study of severely depressed patients undergoing ECT, appreciation scores were high overall with only partial reduction seen in a subgroup with active psychotic symptoms. Physicians judged incapacity in 21.9% of inpatients referred for ECT in Bean et al. (1994/6). This suggests a measurement problem.

Deficits in appreciation ability are reported in both empirical and clinical ethical literature. Some empirical studies additionally report deficits in understanding and reasoning ability. Appelbaum & Grisso (1995 & 1999), Cohen et al. (2004), and Vollman et al. (2003) , all identify understanding and reasoning deficits, with the latter two studies indicating reasoning as the most impaired ability amongst their depressed cohort. Despite these findings, consideration of reasoning ability in relation to DMC is absent from clinical ethical analyses. This may reflect ethical concern that the four abilities model remains too cognitive [4], and that appreciation is the only ability seemingly broad enough to accommodate affective status when judging DMC. Whilst the clinical ethical analyses are well positioned to interpret complex interplay between affect and decision-making abilities it may be that in emphasizing “non-cognitive” abilities in depression, e.g. appreciation, they neglect the impact affect can have on abilities to recall accurately and manipulate information logically. However, the empirical studies that report reasoning impairments more than appreciation impairments are limited by difficulties in the operationalizing of MacCAT-T appreciation (see below). Together these factors could explain why the clinical ethical data and the empirical data show some divergence.

MacCAT appreciation derived from US court rulings during a time when the right to refuse anti-psychotic medication was an important legal issue. Many of these rulings are fairly silent on the detail of appreciation but there is some detail in cases involving schizophrenia where delusions and lack of insight (awareness of illness) were features [27]. Appreciation and insight are often mentioned together. Owen et al. (2009) and Owen et al. (2011) provide evidence that insight is a good indicator of DMC in schizophrenia but a poor one in depression. Grisso and Appelbaum, reflecting on their design of the MacCAT, acknowledge the difficulties they had operationalising appreciation in depression [28]. How depression may impact on the acknowledgement of treatment options, though recognised, is fairly unspecified in the MacCAT-T scoring rules.^b^ .MacCAT appreciation is thus probably better at measuring what is important in schizophrenia than in depression. This may fit with a societal concern not to make the capacity test too sensitive in order to protect liberty interests of patients. Liberty interests, however, need to be balanced against health interests.

Sullivan & Youngner (1994) highlight the demands in assessing appreciative ability; when asking a patient to describe the clinical facts presented, grappling with the pro’s and cons to his or her own life may pose no problem, but when evaluating the patient’s answers to these questions, an assessor may need considerable contextual information and interpretative ability. This might give rise to mis-scoring of the appreciation component of the MacCAT when the contextual and interpretative demands are great – demands which may be high in depressive illness because of the often understandable quality of patients’ decision-making.

Some papers offer potential strategies for dealing with depressed individuals that circumvent assessment of DMC. Rudnick (2002) suggests that consistent treatment preferences should be respected, but, when preferences change, the depressive illness must be alleviated first. Attention has also been drawn to the importance of supported decision-making in which the doctor seeks to enable rather than formally substitute for a depressed patients’ decision–making [22,29,30]. Strategies that attempt to minimize the assessment of DMC offer important, but ultimately unsatisfactory solutions to the ethics of treatment in depressed patients. When depressed individuals change their minds about serious treatments, doctors have to consider how they are going to respect their choices; when patients select harmful choices (e.g. self harm, self neglect) physicians may be unable to support their decisions. At such times the assessment of DMC seems impossible to avoid. An ethically grounded, clinically applicable and reliable framework for the assessment of DMC in depression is thus required.

We found legal judgments on DMC involving cases of depression hard to find – especially those involving treatment or research decisions. In England and Wales where courts have judged DMC in persons with depression findings of lack of DMC for treatment exist [31]. In a case occurring after the introduction of the Mental Capacity Act (2005) [32], a depressed woman’s abilities to decide whether to litigate were explicitly judged. Here, the “use or weigh” ability – an ability that overlaps with “appreciation” – was considered relevant in the final judgment of capacity [33]. A systematic legal search and review of DMC cases involving depression may help clarify the legal perspective.

A limitation of the review is its exclusion of ethical analysis in books and book chapters. Our informal review of leading books in contemporary bioethics revealed little additional attention to the relation between depression and DMC. Elliot [34] devotes a book chapter to it but this draws mainly on his paper in this review. Buchanan and Brock [35] in an endnote refer to how a depressed patient may evidence “indifference” to the harmful consequences of his choice (p392). They take this as evidence for the inadequacy of DMC tests that restrict to an understanding ability. We think it unlikely there is a large literature in book form on depression and DMC we are missing.

## Conclusion

This review highlights a surprisingly small literature on topics of considerable ethical, clinical and policy importance. However, it shows that a start has been made at researching how DMC in depression can be understood, measured and supported. More rigorous clinical ethical studies aiming to interpret and model appreciation, or similar abilities, in depression are needed because most have been based on clinical experience (risking recycling of clinical opinion instead of generation of new knowledge) or case vignettes (risking inadequate data). The law has not articulated its perspective on appreciation, or similar abilities, owing to a lack of depression cases. There is room to improve understanding and measurement of appreciation and progress should be possible as law, clinical ethics and empirical research gains experience of depression.

## Endnotes

^a^Loss of appreciation and reasoning ability are not acknowledged as relevant abilities in making capacity judgements in some states’ law [1] {26}.

^b^When assessing appreciation, the patient is asked whether they think the treatment may benefit them. The patient’s “yes” or “no” response is not important at this point. Instead, the assessor is seeking to establish whether the patients’ explanation regarding their beliefs about treatment/(research) is based on a delusional premise or a serious distortion of reality. However, in recognition of the difficulty faced in the operationalization of appreciation for affective disorder, the MacCAT-T, the scoring rules for appreciation have an additional note*: “failures to acknowledge the potential benefit of treatment may obtain a 0 rating****not only****if they are based on delusional belief systems, but also if they are strongly influenced by extremes in affective symptoms: e.g. mania, severe depression.”* (bold added). ([1] p106 and p184-7).

## Competing interests

The authors declare no conflict of interests.

## Authors’ contributions

TH helped design the study, conducted the literature search analysed the results and drafted the manuscript. MH drafted the manuscript. GO designed the study, supervised the literature search, analysed and checked results and drafted the manuscript. All authors read and approved the final manuscript.

## Pre-publication history

The pre-publication history for this paper can be accessed here:

http://www.biomedcentral.com/1472-6939/14/54/prepub
